# Proposals for improving maternal safety (2023 edition): Insights from the analysis of maternal deaths in Japan

**DOI:** 10.1111/jog.16244

**Published:** 2025-03-11

**Authors:** Japan Maternal Death Exploratory Committee, Murasaki Aman, Tatsuya Arakaki, Masayuki Endo, Junichi Hasegawa, Koji Hashii, Masako Hayashi, Yuki Hosokawa, Tomoaki Ikeda, Hiroshi Ishikawa, Isamu Ishiwata, Chizuko A Kamiya, Takeshi Kanagawa, Naohiro Kanayama, Rie Kato, Shinji Katsuragi, Takao Kobayashi, Tomomi Kotani, Takahiko Kubo, Kentaro Kurasawa, Yoshiki Maeda, Takahide Maenaka, Shintaro Makino, Hiroshi Matsumoto, Kiyonori Miura, Takeshi Murakoshi, Akihito Nakai, Masamitsu Nakamura, Masahiko Nakata, Masafumi Nii, Toshiyuki Okutomi, Kazuhiro Osato, Yoko Sagara, Atsushi Sakurai, Shoji Sato, Akihiko Sekizawa, Yumi Shina, Hiroyuki Sumikura, Toshihito Suzuki, Yoshiyuki Tachibana, Jun C Takahashi, Mayumi Takano, Satoru Takeda, Noboru Tanabe, Kayo Tanaka, Hiroaki Tanaka, Katsuo Terui, Yusuke Todo, Nobuya Unno, Tomoko Wakasa, Tomoyuki Yamashita, Takaaki Yasuda, Jun Yoshimatsu

**Affiliations:** ^1^ Department of Obstetrics and Gynecology Mie University School of Medicine Tsu Mie Japan

**Keywords:** epidural analgesia, group A streptococcal infection, maternal death, pathological autopsy, suicide

## Abstract

The maternal mortality rate remains approximately 4 per 100 000 deliveries. Between January 2010 and July 2024, 629 maternal deaths were reported, of which 590 were reviewed. The Maternal Safety Proposal summarizes these cases. Deaths from obstetric hemorrhage decreased from 28% in 2010 to 7% in 2019 but rose to approximately 20% in 2022 and dropped to 10% in 2023. In the past 4 years, suicide has surpassed obstetric hemorrhage as a leading cause of death. In 2023, intracranial hemorrhage/infarction became the leading cause, with six cases reported. Cardiopulmonary collapse from amniotic fluid embolism, along with cardiovascular, infectious, and pulmonary diseases, has remained stable at 6%–10%. Initial symptoms leading to death occurred during the antepartum (38%), intrapartum (41%), or postpartum periods (21%), with cases distributed across general hospitals, small maternity delivery facilities, and non‐medical settings, including homes. The following are the year's maternal safety proposals:

1. For pregnant women experiencing anxiety, a comprehensive evaluation addressing biological, psychological, and social factors should be conducted to identify key problem areas.
2. Epidural analgesia during labor carries the risk of serious complications. Obstetricians and anesthesiologists must be aware of these risks to ensure the proper management of anesthesia and delivery.
3. Pregnant and postpartum women are at a high risk of invasive group A streptococcal infections, and early screening and timely intervention should be prioritized.
4. Pathological autopsy remains the most effective method for determining the cause of death and should be recommended to bereaved families in all cases of maternal death.

Proposals for Improving Maternal Safety (2023 Edition), created by the Japan Maternal Death Exploratory Committee (JMDEC) and the Japan Association of Obstetricians and Gynecologists (JAOG), was released in September 2024.^1^ An English‐language version of these proposals was created based on the contents of the original Japanese‐language version.

## MATERNAL DEATH REPORT SYSTEM AND CURRENT TREND

In 2010, the JAOG established the JMDEC and a maternal death registration system to enhance the quality of obstetric healthcare and ultimately prevent maternal death. Attending physicians are required to submit detailed reports on maternal deaths to the JAOG, along with medical records, such as anesthesia records, medical images, laboratory data, and pathological and autopsy reports. The JAOG anonymizes these reports before sending them to the JMDEC, which reviews and analyzes the data to identify factors associated with maternal mortality and the circumstances surrounding each death. The JMDEC comprises a multidisciplinary team of obstetricians, gynecologists, anesthesiologists, forensic physicians, emergency physicians, pathologists, psychiatrists, internists, neurosurgeons, lawyers, and other professionals. After a comprehensive discussion, a causal analysis report was prepared for each case, outlining the most probable cause of death and offering recommendations. Finally, the JAOG sent the report back to the submitting institution.^2^, ^3^

**FIGURE 1:**
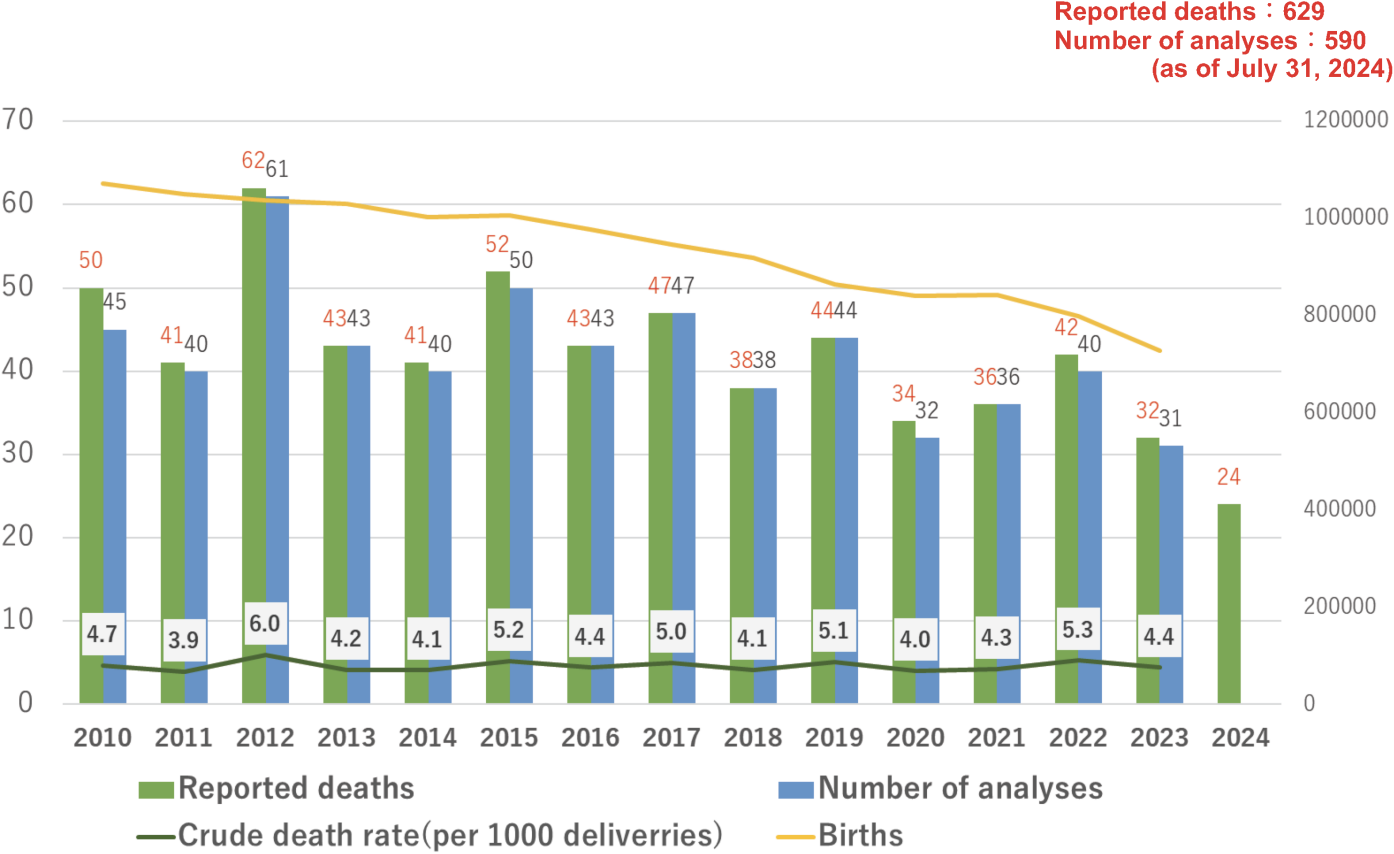
Annual changes in the number of maternal deaths and the number of death reports.

The maternal mortality rate remains approximately 4 per 100 000 deliveries. In total, 629 cases were reported to the project over a 14.5‐year period (from January 2010 to July 2024), of which 590 were reviewed (Figure 1). This proposal for maternal safety reports provides a summary of the cases for which reports have been completed.

## ANNUAL CHANGES IN THE CLASSIFICATION OF CAUSES OF DEATH AMONG PREGNANT WOMEN

**FIGURE 2:**
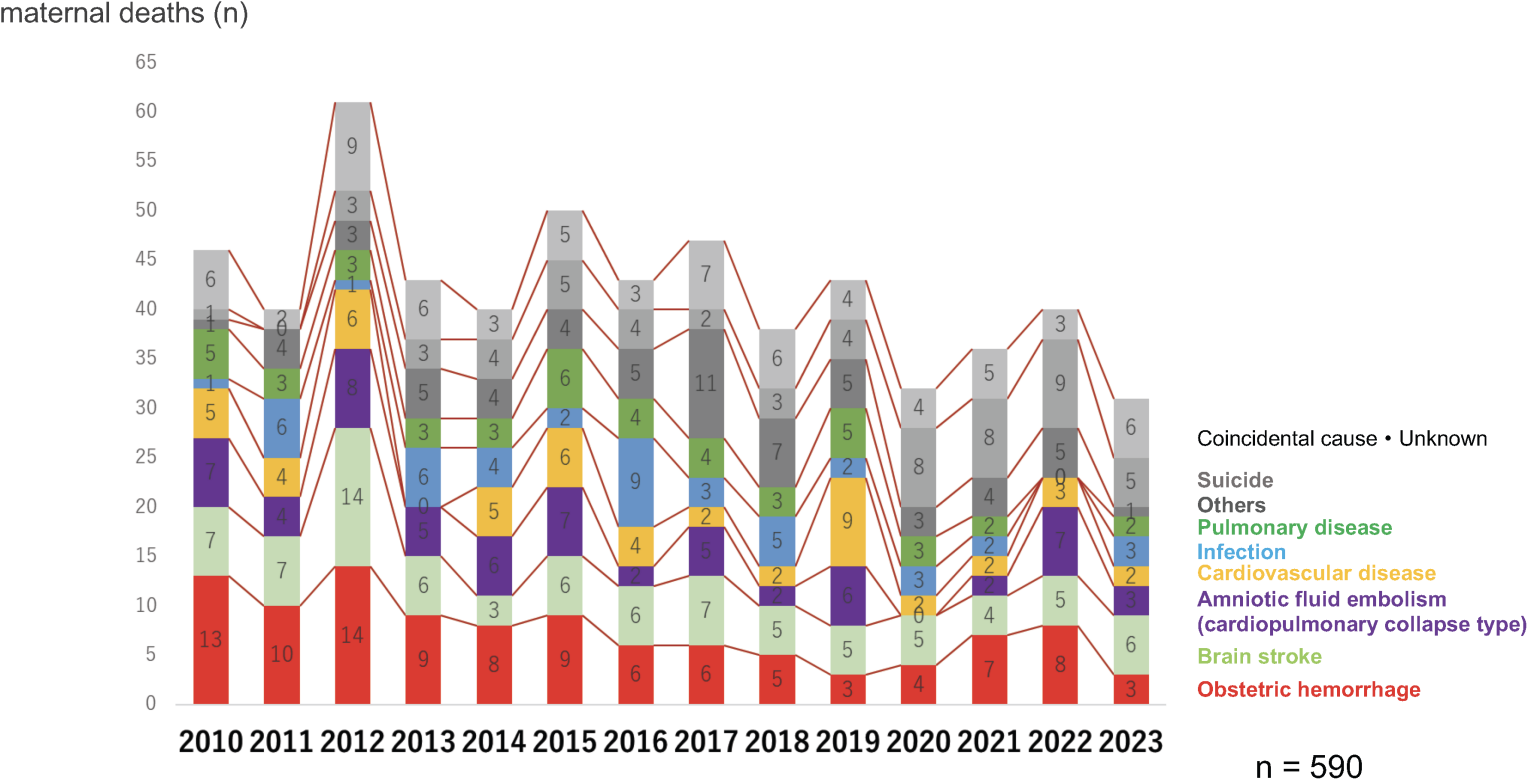
The annual change of the cause of maternal death (ratio of all deaths).

**FIGURE 3:**
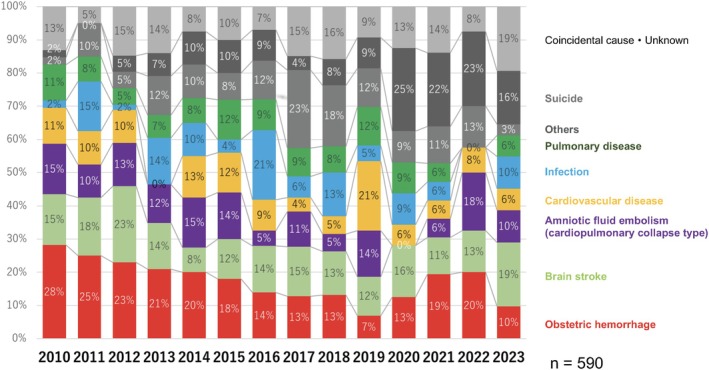
The annual change of the cause of maternal death (real number).

The proportion of deaths due to obstetric hemorrhage, which was approximately 28% in 2010, gradually declined over the years, reaching 7% in 2019 (Figure 2). However, deaths from obstetric hemorrhage have increased again since 2020, peaking at approximately 20% by 2022. By 2023, the rate will decrease by 10%. Over the past 4 years, deaths due to suicide have surpassed those due to obstetric hemorrhage. In 2023, intracranial hemorrhage/infarction emerged as the leading cause of death, with six cases reported (Figure 3). Of these, two were associated with pregnancy‐induced hypertension and four were unrelated. The proportion of deaths due to cardiopulmonary collapse from amniotic fluid embolism, cardiovascular disease, infectious disease, and pulmonary disease has remained stable, ranging between 6% and 10%.

## ONSET TIME AND PLACE OF MATERNAL DEATH

**FIGURE 4:**
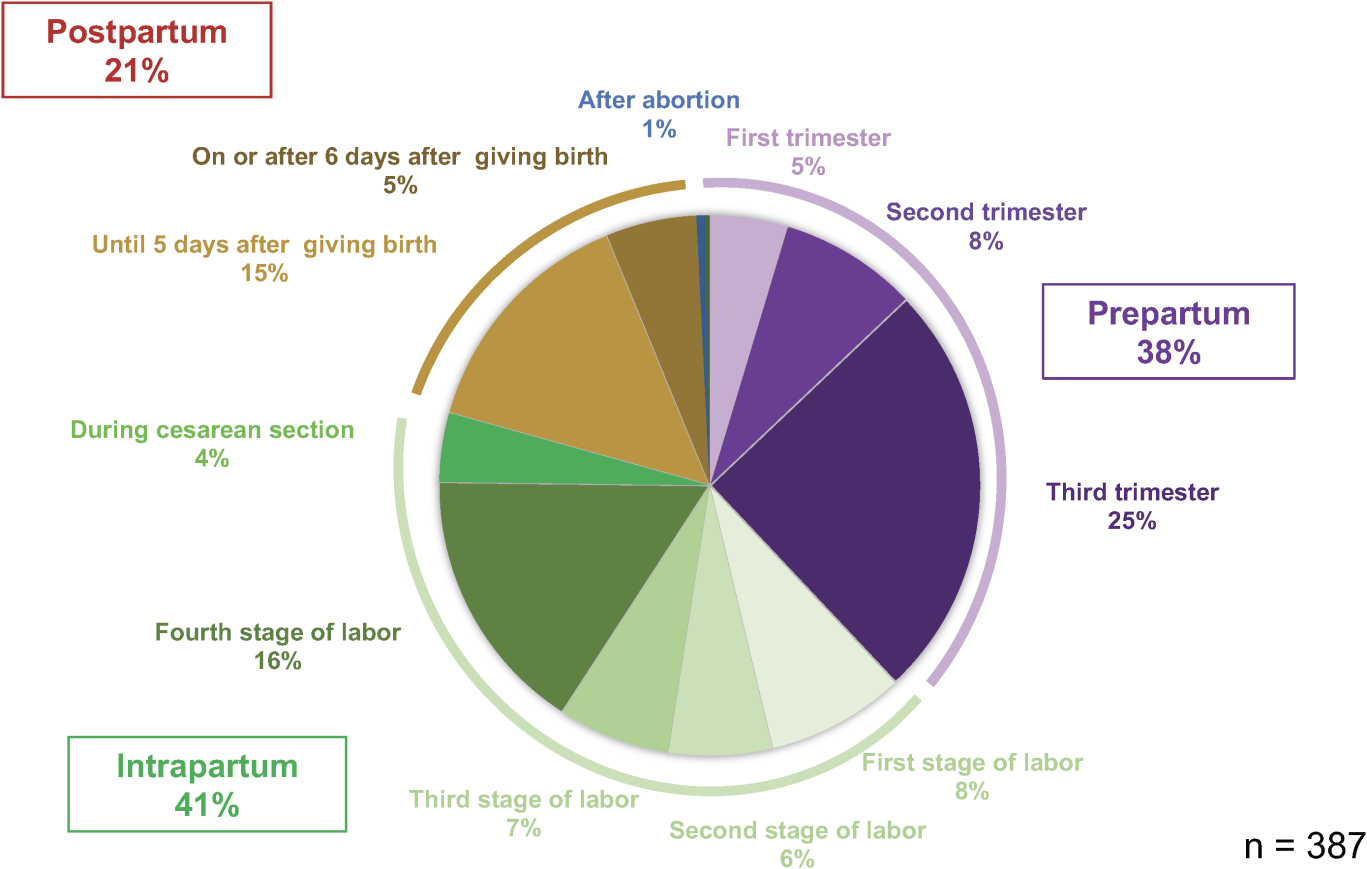
Time of onset of initial symptoms of maternal death.

When classified according to the time of onset of the initial symptoms that led to death, 38% of the cases occurred during pregnancy before delivery began, 41% during delivery, including planned cesarean sections, and 21% during the postpartum period (Figure 4).

**FIGURE 5:**
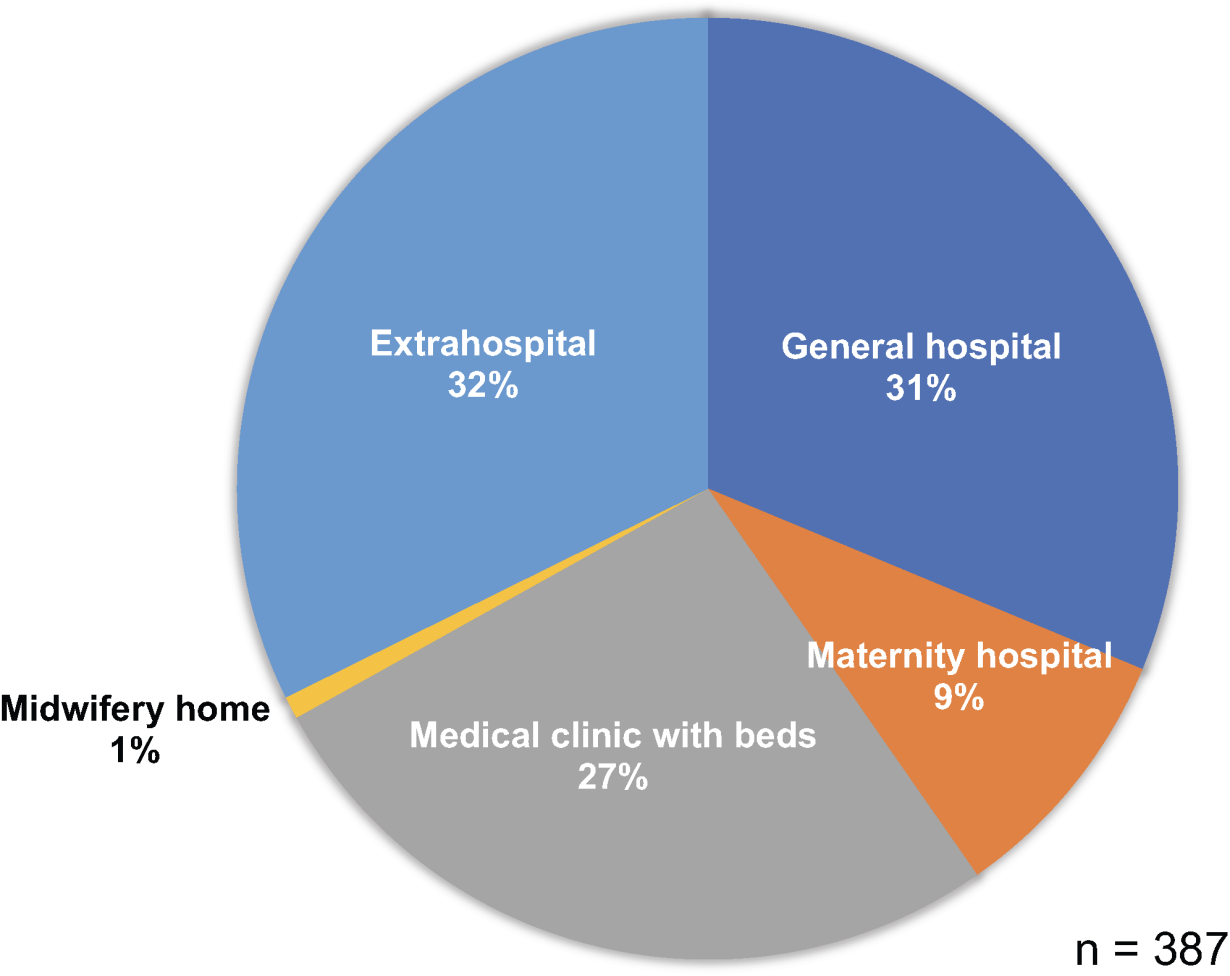
Place of onset of initial symptom of maternal death.

The initial symptoms of pregnancy‐related deaths can occur anywhere and at any time, with one‐third occurring in general hospitals, one‐third in maternity hospitals and clinics with beds, and one‐third outside facilities, including homes (Figure 5).

### Proposal 1

For pregnant women experiencing anxiety, we conducted a comprehensive evaluation addressing biological, psychological, and social factors to identify key problem areas.

#### Case 1

The patient was in her 40s and multiparous. She committed suicide by hanging herself 5 weeks after delivery. She had no history of mental disorders before or during pregnancy. Due to her first child's bronchial asthma, which required frequent visits to the general hospital, she returned to her parents' home and began living with her mother. During delivery at 37 weeks of gestation, an emergency cesarean section was performed because of fetal distress, and the infant was admitted to the NICU. The EPDS score 7 days postpartum was 7. At the 1‐month follow‐up, the mother and child visited the hospital together, and she expressed anxiety about caring for the child; however, it was judged that the mother's mental status was generally stable.

##### Case study

The mother expressed anxiety at her 1‐month postpartum checkup but was not referred for psychiatric care because her condition was not considered severe. After 8 days, the patient committed suicide. This highlights the importance of recognizing anxiety as a potential symptom of postpartum depression, especially when additional stressors, such as a child's illness (asthma in older children and NICU admission in younger children), are present.

##### Comment

In Japan, suicide accounted for 22%–26% of maternal deaths from 2020 to 2022, often involving women who did not receive psychiatric care because their symptoms were mild. It is crucial to provide ongoing support to pregnant women who express anxiety, as anxiety may worsen rapidly and lead to suicidal thoughts. Psychotic symptoms, self‐blame, aversion toward children, and suicidal ideation should prompt immediate care.^4^, ^5^

Therefore, pregnant and postpartum women should be assessed from a biopsychosocial perspective (Table 1). Mood swings in the early postpartum period, often linked to hormonal changes (Bio), require careful observation without hasty conclusions. Although referral to a psychiatrist may not always be necessary, evaluating a patient's mental state, concerns, and potential suicidal thoughts is crucial (psycho). A comprehensive approach should also include interviewing key figures like the mother and husband to offer support (social). Viewing anxiety and depression in this context requires understanding the biological, psychological, and social factors to provide appropriate care and treatment.

**TABLE 1:** Examples of biopsychosocial aspects to consider when understanding maternal mental health.

Aspects to evaluate	Example
Biological aspects	Rapid hormonal fluctuations during pregnancy and postpartum periods Physical changes associated with pregnancy and childbirth Mood swings and irritability in the early postpartum period
Psychological aspects	Growth history Personality predisposition Presence of mental illness Presence of suicidal ideation Course and specific details of anxiety
Social aspects	Family background, relationships, and key person Childcare situation Child's health and upbringing environment (including children other than the child of this pregnancy and delivery) Status of support within the family for housework and childcare Social isolation Financial problems

### Proposal 2

Labor epidural analgesia can lead to serious complications; therefore, both obstetricians and anesthesiologists should be aware of these complications and provide appropriate analgesia/delivery management:

1. Maintain epidural analgesia with a combination of a low‐concentration local anesthetic and fentanyl.^6^
2. Epidural analgesia can mask pain and other symptoms caused by uterine rupture and retroperitoneal hemorrhage. Close assessment of vital signs and bleeding is needed.

#### Case 2

The patient was in her 20s and a primipara. Her pre‐pregnancy BMI was 30. At 40 weeks of gestation, she presented with premature rupture of the membranes and was administered epidural analgesia. The patient was unable to move her lower limbs because of epidural analgesia. During vacuum extraction, she lost consciousness and experienced seizures, followed by cardiac arrest. She was transferred to a higher‐level facility but died. A pathological autopsy revealed a pulmonary embolism. The duration of labor was 70 h, and the duration of epidural analgesia was 40 h.

##### Case study

The cause of maternal death was pulmonary thromboembolism (PTE), which can be attributed to obesity and prolonged motor blockade in the lower limbs. Motor blockade was induced by 0.25% bupivacaine, which is used for the maintenance of labor epidural analgesia. Additionally, prolonged labor (70 h) without timely labor‐enhancing interventions may be a key factor in the development of PTE.

#### Case 3

The patient was in her 30s and a multipara. She received epidural anesthesia at 39 weeks of gestation for a pain‐free delivery. After anesthetics and fentanyl were administered, she delivered a 3300 g baby via vacuum extraction with Kristeller fundal pressure. Postdelivery, mild back pain led to ketamine, midazolam, and ephedrine administration during perineal suturing. After 2 h, she became hypotensive (60/25 mmHg) and tachycardic (160 bpm), prompting a hospital transfer. Despite minimal genital bleeding, ultrasonography revealed the presence of abdominal fluid. The patient did not respond to resuscitation. Cause of death: uterine rupture near the uterine artery.

##### Case study

This patient died from critical hemorrhage due to intra‐abdominal bleeding from a uterine rupture. Rapid delivery likely triggered rupture, but painless delivery may mask symptoms and delay detection. Despite the lack of clear symptoms, postdelivery shock warrants immediate investigation, including ultrasonography.

##### Comment

**FIGURE 6:**
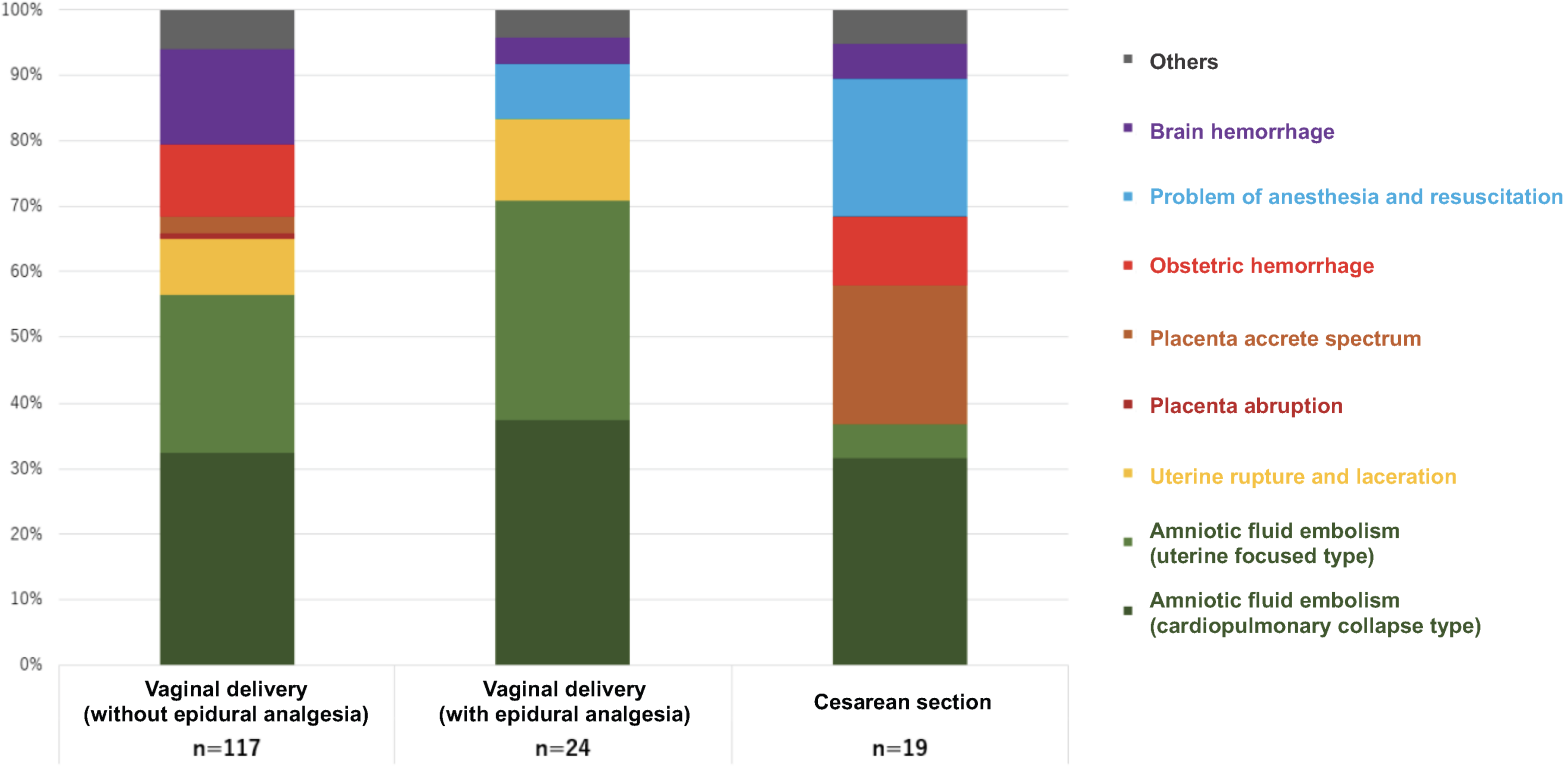
Cause of death in 160 cases of maternal death that occurred during childbirth.

Of the maternal deaths analyzed by the JMDEC, 160 occurred during childbirth, with 24 cases (15%) involving vaginal deliveries with epidural analgesia (Figure 6). Among these, there was one case of high spinal subarachnoid anesthesia and one case of local anesthetic poisoning directly linked to the anesthesia. Additionally, 17 patients (71%) developed amniotic fluid embolism: 9 with cardiopulmonary collapse and 8 with the uterine‐focused type. There were also three cases (12%) involving uterine and birth canal lacerations, all associated with vacuum delivery, with the Kristeller uterine fundal pressure maneuver used in two cases. Of the 24 epidural analgesia cases, 23 (96%) involved planned labor induction, suggesting that deaths such as amniotic fluid embolism and uterine rupture may be more strongly linked to planned labor induction than to the analgesia per se.

### Proposal 3

Recognize that pregnant and postpartum women are at high risk for invasive group A streptococcal infections and prioritize early screening and timely intervention.

#### Case 4

A multiparous woman in her 30s visited her family doctor with fever at 37 weeks of gestation. She was prescribed acetaminophen and sent home. Later, in the afternoon, she experienced heavy bleeding and abdominal pain, prompting a visit to a small maternity facility. The infant was born in the evening. Her vital signs were abnormal, including a low blood pressure, and she was transferred to a high‐level facility. Despite intensive treatment, she developed multiple organ failure and died 30 h after transfer. Administration of FMOX was delayed by 24 h. Pathological examination of the uterus revealed bacterial colonies in the necrotic myometrium and blood clots in the blood vessels. *Streptococcus pyogenes* was detected in the uterine cavity, leading to the diagnosis of an invasive group A streptococcal infection.

##### Case study

This case involved a stillbirth due to severe uterine contractions from an invasive Group A streptococcal infection, leading to death from septic shock and DIC. Upper respiratory symptoms, which were likely the source of infection, were present. The family doctor did not consider Group A streptococcal infection during the initial examination for respiratory symptoms. Upon transportation, her vital signs (qSOFA score) were suggestive of sepsis. The CENTOR criteria and rapid antigen tests should be used to confirm the diagnosis of Group A streptococcal infections.

##### Comment

Pregnant or postpartum patients have a 20‐fold increased incidence of GAS infection compared with non‐pregnant individuals.^7^ Among women of childbearing age (15–44 years), 20% of invasive GAS infections are pregnancy related.^8^ A recent systematic review found that most GAS infections (91.9%) occur postpartum, 4.7% occur antepartum, and 0.6% occur intrapartum,^9^ in contrast to the antenatal dominance of deaths (71%) in Japan.

Of the 616 maternal deaths between 2010 and March 2024, 48 (8%) were caused by infectious diseases, with invasive GAS being the most common cause (56%, *n* = 27). Of these, 21 occurred during antepartum and 6 occurred during postpartum. In 71% (15/21) of the antepartum GAS‐toxic shock syndrome (TSS) cases, the infection originated from the upper respiratory tract, whereas 67% (4/6) of the puerperium cases involved genital tract infections. It should be emphasized that the main route of infection for intensive GAS cases in the antenatal period is through the upper respiratory tract.^10^

Notably, no maternal deaths due to GAS‐TSS have been reported during the COVID‐19 pandemic in Japan (2020–2023).^11^ These findings suggest that preventive measures, such as frequent disinfection, wearing masks, and isolation from high‐risk individuals, such as symptomatic children, may help reduce antepartum GAS infections.

Example of Outpatient Prescription for Group A Streptococcal Infection:

- Amoxicillin (such as Pasetocin®, Sawacillin®): 750–1500 mg, three times a day, for 10 days
- Cephalexin (such as Keflex®): 1000 mg, four times a day, for 7 days.

### Proposal 4

Pathological autopsy remains the most effective way to determine the cause of death and should be explained to the bereaved families of all maternal death cases.

#### Case 5

A woman in her 40s, a primipara, presented at 34 weeks of pregnancy with headache and vomiting upon waking. Her blood pressure was 170/110 mmHg. Chest radiography revealed pulmonary edema, and echocardiography revealed severe cardiac dysfunction with an LVEF of <20%. An emergency cesarean section was performed. Despite the initial improvement in heart function, she suffered cardiopulmonary arrest 12 h later, and despite resuscitation efforts, was pronounced dead. Postmortem examination revealed an adrenal mass, confirming the diagnosis of pheochromocytoma.

##### Case study

This case underscores the critical importance of autopsies in uncovering diagnoses missed during a patient's lifetime. In this instance, the autopsy revealed pheochromocytoma as the underlying cause of secondary hypertension and acute heart failure, providing crucial insights into the cause of death that might have otherwise remained unidentified.

##### Comment

**FIGURE 7:**
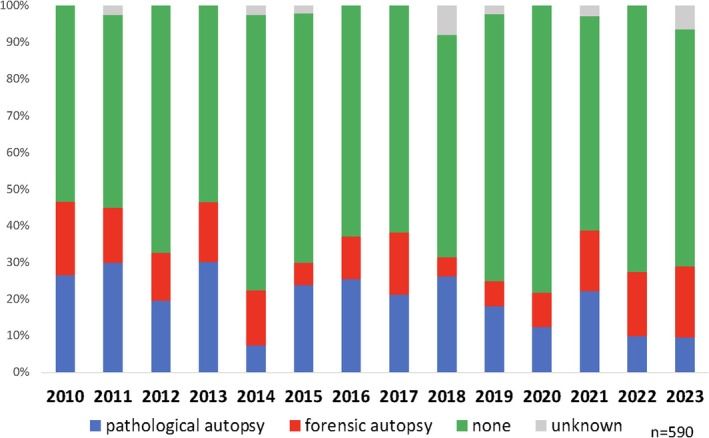
Trends in the autopsies rate conducted in cases of maternal death.

In 2010, the autopsy rate for maternal deaths was approximately 50% and was split between pathological and judicial autopsies (Figure 7). Recently, this rate has dropped to approximately 30% with fewer pathological autopsies. In 2023, only 3 of 31 maternal deaths underwent pathological autopsy.

Among the 505 maternal deaths between 2012 and 2023, 162 underwent autopsies. In 72 cases (44%), the clinical and autopsy diagnoses matched. Autopsies revealed a different cause of death in 14 cases (9%), and a diagnosis was ruled out in 8 cases (5%). Additionally, 24 (15%) patients showed new findings that were not clinically detected. Overall, autopsies provided useful insights in 29% of the cases. Despite advancements in imaging, autopsies remain crucial, especially for diagnosing conditions such as fulminant type 1 diabetes and pheochromocytoma, in which organ changes are subtle.^12^, ^13^

## AUTHOR CONTRIBUTIONS

**Tomoaki Ikeda:** Conceptualization; methodology; project administration; writing – original draft; writing – review and editing.

## CONFLICT OF INTEREST STATEMENT

The authors declare no conflict of interest for this article.

## APPENDIX A Japan Maternal Death Exploratory Committee members

The members of the Japan Maternal Death Exploratory Committee include Murasaki Aman, Tatsuya Arakaki, Masayuki Endo, Junichi Hasegawa, Koji Hashii, Masako Hayashi, Yuki Hosokawa, Tomoaki Ikeda, Hiroshi Ishikawa, Isamu Ishiwata, Chizuko A Kamiya, Takeshi Kanagawa, Naohiro Kanayama, Rie Kato, Shinji Katsuragi, Takao Kobayashi, Tomomi Kotani, Takahiko Kubo, Kentaro Kurasawa, Yoshiki Maeda, Takahide Maenaka, Shintaro Makino, Hiroshi Matsumoto, Kiyonori Miura, Takeshi Murakoshi, Akihito Nakai, Masamitsu Nakamura, Masahiko Nakata, Masafumi Nii, Toshiyuki Okutomi, Kazuhiro Osato, Yoko Sagara, Atsushi Sakurai, Shoji Sato, Akihiko Sekizawa, Yumi Shina, Hiroyuki Sumikura, Toshihito Suzuki, Yoshiyuki Tachibana, Jun C Takahashi, Mayumi Takano, Satoru Takeda, Noboru Tanabe, Kayo Tanaka, Hiroaki Tanaka, Katsuo Terui, Yusuke Todo, Nobuya Unno, Tomoko Wakasa, Tomoyuki Yamashita, Takaaki Yasuda, and Jun Yoshimatsu.

### Affiliations

Miyazaki University School of Medicine, Miyazaki, Japan; Showa University School of Medicine, Tokyo, Japan; Osaka University Graduate School of Medicine, Osaka, Japan; St. Marianna University School of Medicine, Kanagawa, Japan; Hashii Women's Hospital, Kyoto, Japan; Nippon Medical School Tama Nagayama Hospital, Tokyo, Japan; Mie University School of Medicine, Mie, Japan; Kanagawa Children's Medical Center, Kanagawa, Japan; Ishiwata Obstetrics and Gynecology Hospital, Ibaraki, Japan; National Cerebral and Cardiovascular Center, Osaka, Japan; Shizuoka College of Medical Care Science, Shizuoka, Japan; Hamamatsu Medical Center, Shizuoka, Japan; Nagoya University Graduate School of Medicine, Aichi, Japan; Shirota Obstetrics and Gynecological Hospital, Kanagawa, Japan; Yokohama City University School of Medicine, Kanagawa, Japan; Kuwana City Medical Center, Mie, Japan; Higashiosaka City Medical Center, Osaka, Japan; Urayasu Hospital, Juntendo University, Chiba, Japan; Nagasaki University Graduate School of Biomedical Sciences, Nagasaki, Japan; Seirei Hamamatsu General Hospital, Shizuoka, Japan; Aiiku Maternal and Child Health Center, Tokyo, Japan; Fujita Health University, Aichi, Japan; Toho University Omori Medical Center, Tokyo, Japan; Kitazato University Hospital, Kanagawa, Japan; Mie Prefectural General Medical Center, Mie, Japan; Nihon University School of Medicine, Tokyo, Japan; Oita Prefectural Hospital, Oita, Japan; St. Luke's International Hospital, Tokyo, Japan; Juntendo Koshigaya Hospital, Juntendo University Faculty of Medicine, Saitama, Japan; Tokyo Metropolitan Chubu General Mental Health Welfare Center, Tokyo, Japan; Kindai University Faculty of Medicine, Osaka, Japan; Juntendo University School of Medicine, Tokyo, Japan; Nakamura Hirai Tanabe Law Office, Osaka, Japan; JCHO Kumamoto General Hospital, Kumamoto, Japan; Saitama Medical Center, Saitama Medical University, Saitama, Japan; Hamamatsu University School of Medicine, Shizuoka, Japan; JCHO Sagamino Hospital, Kanagawa, Japan; Kindai University Nara Hospital, Nara, Japan; Japanese Red Cross Medical Center, Tokyo, Japan.

## References

[1] Japan Maternal Death Exploratory Committee , Japan Association of Obstetricians and Gynecologists . Proposals for Improving Maternal Safety (2023 Edition) in Japanese. 2024 Available at https://www.jaog.or.jp/wp/wp-content/uploads/2023/10/botai_2023.pdf Accessed 6 Feb 2025.

[2] Hasegawa J , Katsuragi S , Tanaka H , Kurasaki A , Nakamura M , Murakoshi T , et al. Decline in maternal death due to obstetric haemorrhage between 2010 and 2017 in Japan. Sci Rep. 2019;9:11026. 31363105 10.1038/s41598-019-47378-z PMC6667693

[3] Hasegawa J , Sekizawa A , Tanaka H , Katsuragi S , Osato K , Murakoshi T , et al. Current status of pregnancy‐related maternal mortality in Japan: a report from the Maternal Death Exploratory Committee in Japan. BMJ Open. 2016;6:e010304. 10.1136/bmjopen-2015-010304 PMC4809072 27000786

[4] Patterson WM , Dohn HH , Bird J , Patterson GA . Evaluation of suicidal patients: the SAD PERSONS scale. Psychosomatics. 1983;24:343–349. 6867245 10.1016/S0033-3182(83)73213-5

[5] Knight M , Bunch K , Tuffnell D , Jayakody H , Shakespeare J , Kotnis R , et al. Saving lives, improving Mothers' Care ‐ lessons learned to inform maternity care from the UK and Ireland confidential enquiries into maternal deaths and morbidity 2014–2016. Oxford: National Perinatal Epidemiology Unit, University of Oxford; 2018.

[6] Wong CA . Epidural and spinal analgesia: anesthesia for labor and vaginal delivery. In: Chestnut DH, Wong CA, Tsen LC, Ngan Kee WD, Beilin Y, Mhyre JM et al., editors. Obstetric anesthesia. 6th ed. Philadelphia: Elsevier; 2020.

[7] Deutscher M , Lewis M , Zell ER , Taylor TH , van Beneden C , Schrag S , et al. Incidence and severity of invasive *Streptococcus pneumoniae*, group A *Streptococcus*, and group B *Streptococcus* infections among pregnant and postpartum women. Clin Infect Dis. 2011;53:114–123. 21690617 10.1093/cid/cir325

[8] Nelson GE , Pondo T , Toews KA , Farley MM , Lindegren ML , Lynfield R , et al. Epidemiology of invasive group A streptococcal infections in the United States, 2005‐2012. Clin Infect Dis. 2016;63:478–486. 27105747 10.1093/cid/ciw248 PMC5776658

[9] Harris K , Proctor LK , Shinar S , Philippopoulos E , Yudin MH , Murphy KE . Outcomes and management of pregnancy and puerperal group A streptococcal infections: a systematic review. Acta Obstet Gynecol Scand. 2023;102:138–157. 36636775 10.1111/aogs.14500 PMC9889326

[10] Hasegawa J , Sekizawa A , Tanaka H , Katsuragi S , Tanaka K , Nakata M , et al. Infection route associated with invasive group A streptococcal toxic shock syndrome in maternal deaths: Nationwide analysis of maternal mortalities in Japan. Int J Infect Dis. 2024;146:107154. 38936654 10.1016/j.ijid.2024.107154

[11] Hasegawa J , Tanaka H , Katsuragi S , Nii M , Sekizawa A , Ishiwata I , et al. Maternal death due to serious group A streptococcal toxic shock syndrome reduced after the coronavirus disease pandemic in Japan. J Matern Fetal Neonatal Med. 2022;35:10451–10454. 36195456 10.1080/14767058.2022.2128663

[12] Tanaka H , Katsuragi S , Hasegawa J , Tanaka K , Osato K , Nakata M , et al. The most common causative bacteria in maternal sepsis‐related deaths in Japan were group a *Streptococcus*: a nationwide survey. J Infect Chemother. 2019;25(1):41–44. 10.1016/j.jiac.2018.10.004 30377069

[13] Hayata E , Nakata M , Hasegawa J , Tanaka H , Murakoshi T , Mitsuda N , et al. Nationwide study of mortality and survival in pregnancy‐related streptococcal toxic shock syndrome. J Obstet Gynaecol Res. 2021;47:928–934. 33350021 10.1111/jog.14619

